# Long-term maintenance of synaptic plasticity by Fullerenol Ameliorates lead-induced-impaired learning and memory in vivo

**DOI:** 10.1186/s12951-022-01550-2

**Published:** 2022-08-01

**Authors:** Yingying Zha, Yan Jin, Xinxing Wang, Lin Chen, Xulai Zhang, Ming Wang

**Affiliations:** 1 Department of Otolaryngology-Head and Neck Surgery, The First Affiliated Hospital of USTC, Division of Life Sciences and Medicine, University of Science and Technology of China, Hefei, 230001 Anhui China; 2 Cell Electrophysiology Laboratory, Wannan Medical College, Wuhu, 241002 Anhui China; 3 Stroke Center and Department of Neurology, The First Affiliated Hospital of USTC, Division of Life Sciences and Medicine, University of Science and Technology of China, Hefei, 230036 Anhui China; 4 Hefei National Laboratory for Physical Sciences at Microscale, and School of Life Sciences, University of Science and Technology of China, Hefei, 230027 Anhui China; 5 Biomedical Sciences and Health Laboratory of Anhui Province, University of Science and Technology of China, Hefei, 230027 Anhui China; 6 Department of Medical Education and Research, Anhui Clinical Center for Mental and Psychological Diseases, Hefei Fourth People’s Hospital, Hefei, 230022 Anhui China

**Keywords:** Fullerenol, Lead-induced impairment, Synaptic plasticity, Learning and memory, In vivo

## Abstract

Fullerenol, a functional and water-soluble fullerene derivative, plays an important role in antioxidant, antitumor and antivirus, implying its enormous potential in biomedical applications. However, the in vivo performance of fullerenol remains largely unclear. We aimed to investigate the effect of fullerenol (i.p., 5 mg/kg) on the impaired hippocampus in a rat model of lead exposure. Matrix-assisted laser desorption/ionization time-of-flight mass spectrometry (MALDI-TOF–MS) is a kind of newly developed soft-ionization mass spectrometry technology. In the present study, an innovative strategy for biological distribution analysis using MALDI-TOF–MS confirmed that fullerenol could across the blood–brain barrier and accumulate in the brain. Results from behavioral tests showed that a low dose of fullerenol could improve the impaired learning and memory induced by lead. Furthermore, electrophysiology examinations indicated that this potential repair effect of fullerenol was mainly due to the long-term changes in hippocampal synaptic plasticity, with enhancement lasting for more than 2–3 h. In addition, morphological observations and biochemistry analyses manifested that the long-term change in synaptic efficacy was accompanied by some structural alteration in synaptic connection. Our study demonstrates the therapeutic feature of fullerenol will be beneficial to the discovery and development as a new drug and lays a solid foundation for further biomedical applications of nanomedicines.

**Supplementary Information:**

The online version contains supplementary material available at 10.1186/s12951-022-01550-2.

## Introduction

Fullerene has high potential in biomedical applications, but the applications are restricted by their extremely poor solubility in polar solvents [1]. Polyhydroxylated fullerene (called fullerenol), produced by adding the OH groups to the carbon surface of fullerene, has greatly improved water-solubility and decreased cytotoxicity [2]. Fullerenol also shows outstanding characteristics including antioxidant activity, antiviral property and anti-inflammatory function in chemical and biological systems [3, 4]. Moreover, some derivatives have the ability to reduce excitotoxicity and apoptosis after epileptic seizures, prevent functional disturbances in hippocampus, inhibit glioblastoma cell proliferation and improve neural regeneration [5–8]. In a word, derivatives of fullerene may act as neuroprotectants in the central nervous system.

Lead does not have an effective physiological role in organisms but has numerous harms [9–11]. Because lead can mimic calcium, disrupt tight junctions, increase permeability surface area and induce brain perfusion, it will target the brain vasculature and damage the blood–brain barrier system [12–14]. Lead poisoning may interfere with normal brain development, including blocking synaptogenesis in the cerebral cortex, reducing the number of neurons, depressing neurotransmission and neuronal growth [15]. In addition, lead has been found to induce neuronal apoptosis in the brain and damage the hippocampus involved in memory processes, which can cause learning disabilities, decreased IQ and behavioral abnormalities [11, 16–19]. In short, the hippocampus may be one of the most sensitive organs to lead toxicity.

Unfortunately, there is no specific cure for lead poisoning, and victims are likely to suffer for life. Our previous study has shown that a low-concentration of fullerenol alleviates the lead-induced impairment of cultured hippocampal neurons through an antioxidant mechanism [20]. However, it is unclear whether the in vivo performance of fullerenol can affect lead-induced-impaired learning and memory. The present study investigated the effect of fullerenol through behavioral tests, electrophysiology examinations, morphological observations and biochemistry analyses, in order to provide a new idea for the treatment of lead poisoning and lay a solid foundation for further biomedical applications of nanomedicines.

## Experimental section

### Nanoparticle preparation and characterization

Fullerenol, [C_60_(OH)_24_], purchased from the Materials and Electrochemical Research Corporation (USA), was dissolved in double-distilled water and stored at 4 °C. The mean diameter and zeta potential of fullerenol in water were monitored by using a professional instrument (Zetasizer Nano ZS90, Malvern Instruments Ltd., UK), and the morphology of fullerenol was characterized by Transmission Electron Microscope (TEM, JEOL-2010, Japan Electron Optics Laboratory Co. Ltd., Japan) at an accelerating voltage of 200 kV.

### Animals and treatments

Wistar rats used in the present study were purchased from Shanghai SLAC Laboratory Animal Co. Ltd. (China). Male rats after birth were randomly divided into four groups. In the control group (n > 8), rats received normal treatments with pure drinking water (free-feeding and free-drinking) and injection of saline (the same volume as fullerenol). In the fullerenol-exposed group (n > 8), rats received similar treatments but with injection of fullerenol (5 mg/kg i.p., every other day after weaning, 12 times, d22-45). In the lead-exposed group (n > 8), rats received modeling treatments with 0.2% lead acetate drinking water (pups were exposed through milk from their mothers) and injection of saline. In the fullerenol-intervene group (n > 8), rats received special treatment with 0.2% lead acetate drinking water and injection of fullerenol.

### Electron microscopy

The image was performed on a field-emission Transmission Electron Microscopy (TEM, FEI Tecnai G2 F20, Hillsboro) operated at 200 kV to determine the physical property of fullerenol.

The number of postsynaptic density (PSD) in the hippocampal CA1 region was also observed using TEM. About 1 mm^3^ of CA1 tissue block was immersed in 1 ml glutaraldehyde for at least 24 h, and photos acquired from randomly selected areas were analyzed by calculating the number of PSD.

### Matrix-assisted laser desorption/ionization time-of-flight mass spectrometry (MALDI-TOF–MS)

The brain, liver, kidney and spleen of a Wistar rat (i.p., 5 mg/kg, last for 24 h) were collected as the analytic samples and 1 mM water-soluble fullerenol was made as the standard. After dissolved in nitric acid, heated for 6 h, purified by acetone and methanol until transparent and colorless, all samples were dissolved in toluene and analyzed using MALDI-TOF–MS (Autoflex Speed TOF, Bruker Corporation, USA).

### Blood lead and hippocampal lead analysis

Fresh blood (about 0.1 mL) from each experimental rat was collected by cardiac puncture in vacuum heparin tube. Meanwhile, hippocampus from each anesthetized and decapitated rat after all experiments was collected, rinsed and weighted. Then excess pure nitric acid was added to react with samples and to dissolve the precipitate. Finally, the mixture was analyzed by an Inductively Coupled Plasma Mass Spectrometer (ICP-MS, X Series 2, Thermo Fisher Scientific, USA) to measure the lead levels. The operations were all performed in ICP-MS lab, which met strict conditions and avoided lead pollution.

### Open field test

The open field test, which reflected spontaneous activity and exploratory movement, was conducted to evaluate the behavioral performance of each experimental rat to ensure its suitability for later tests. A rat was placed in the center of a square enclosure (90 cm × 90 cm) with 16 equal arenas (22.5 cm × 22.5 cm). In a quiet environment, the performance of each rat was tracked for 5 min and analyzed with a professional software (Visu Track, XinRuan, China).

### Morris water maze

In Morris water maze, a small wading pool (160 cm in diameter, 50 cm deep) was filled with water to a depth of 1–2 cm above the surface of a circular escape platform (12 cm in diameter). The animal must learn to navigate a direct path to the hidden platform from different locations using distal cues. During the training period of 5 days, each rat was placed in the pool from a random non-platform quadrant, and given 90 s to locate the platform, and remained on the hidden platform for 30 s. On the sixth day, the rat was placed in the pool without a platform from the quadrant opposite the previous platform position. The behavior was recorded for 2 min by a video camera and analyzed with a tracking software (EthoVision XT 5.0, SHANGHAI BIOWILL CO., LTD, China).

### In vivo field potential recording

The field excitatory postsynaptic potential (fEPSP) was recorded to further evaluate the electrophysiological function of the hippocampus in each anesthetized rat by in vivo field potential recording. In main input pathway of the hippocampus (PP-DG pathway), the stimulating electrode was placed in the lateral perforate fiber and the recording electrode was in the DG area, while in main output pathway (Sch-CA1 pathway), they were placed in the pyramidal cell layer of CA3 and CA1 respectively.

The input–output (I/O) curve reflected the postsynaptic reactions under a series of different electrical stimuli. The stimulus current was from 0.1 to 1.4 mA by steps of 0.1 mA, while frequency and pulse were 0.05 Hz and 0.2 ms respectively. The paired pulse facilitation (PPF) ratio reflected the responses to two stimuli delivered at short inter-stimulus intervals from 10 to 800 ms and was expressed as fEPSP2/fEPSP1. The stimulus intensity was adjusted to 40–60% of the maximal amplitude of the population spike (PS, assessed by the I/O curve). After a 30 min rest break, the baseline recording was obtained for 20 min, and then long-term potentiation (LTP) was elicited by applying a high frequency stimulus (HFS, PP-DG pathway: 250 Hz, 1 s, 11 cycles; Sch-CA1 pathway: 200 Hz, 1 s, 5 cycles, repeated 6 times at intervals of 1 min). The post-HFS recording was performed for 1 h (PP-DG pathway) or 4 h (Sch-CA1 pathway) with a single pulse applied at a frequency of 0.05 Hz. These responses were normalized to baseline values, and the EPSP slope and PS amplitude of each group were averaged every 5 min.

### Golgi staining

A brain block (15 × 15 × 5 mm^3^, containing hippocampus) was immersed in chromic acid mixed solution at 37 °C for 2–3 days. After rinsing with distilled water, the tissue was immediately cut at 150 μm with a vibrating slicer (DTK-1000, MICROSLICER, DSK). And then all slices were dehydrated by a graded series of ethanol (70%, 80%, 95%, 99%) and 99% n-butyl alcohol, developed with ammonia water and sodium thiosulfate, and cleared with xylene. Finally, Golgi-treated slices mounted with neutral resin were imaged by a light microscope (640 ×), and the numbers of spines were analyzed.

### Western blotting analysis

Levels of total CaMKIIα and pCaMKIIα (CaMKII phosphorylated at T286) were investigated in hippocampi of experimental rats (n = 4 for each group). Each hippocampus was homogenized in lysis buffer (pH 7.4, 50 mM Tris, 150 mM NaCl, 1% Triton X-100, 1% sodium deoxycholate, 0.1% SDS, sodium orthovanadate, sodium fluoride, EDTA, leupeptin, etc.) and centrifuged at 12,000*g* for 15 min. After being diluted and denatured, the samples (about 20 μg) were separated by SDS-PAGE (10% resolving gel), transferred to a PVDF membrane and blocked for 1 h. Then the membrane was incubated with mouse polyclonal anti-CaMKII antibody (1:400, Santa Cruz Biotechnology) or rabbit polyclonal anti-pCaMKII antibody (1:400, Santa Cruz Biotechnology) overnight at 4 °C. After incubation with a horseradish peroxidase-coupled secondary antibody (1:3000, Beyotime, China) for 2 h at room temperature, the bands were visualized using the ECL detection system. The value of the band was calculated using the Bio-Rad video imaging system and expressed as a percentage of GAPDH.

### Measurement of oxidative stress

Assay kits obtained from the Beyotime Institute of Biotechnology (China) were used to measure the hydrogen peroxide (H_2_O_2_) level (product id: S0038), the total antioxidant capacity (product id: S0121), the total superoxide dismutase (SOD) activity (product id: S0103) and the glutathione (GSH) concentration (product id: S0052). Briefly, hippocampal specimens were collected and pestled in cold buffered saline. The homogenates were centrifuged at 12,000*g* for 5 min at 4 °C, and the supernatants were stored at − 80 °C and used following the protocols.

H_2_O_2_ oxidizes divalent iron ions to trivalent iron ions, and then forms purple products with xylenol orange, so as to realize the determination of H_2_O_2_ level. The oxidation process of ABTS (a chromogenic agent) is inhibited in the presence of antioxidants, and the total antioxidant capacity can be calculated by absorbance measurement. At present, there are many kinds of SOD activity determination methods, among which WST-8 method is widely used because of better stability and higher sensitivity. WST-8 (a substrate) can react with the superoxide anion to produce formazan dye but be inhibited by SOD, and the activity of SOD can be calculated. Oxidized glutathione is reduced and then reacted with chromogenic substrate, and the yellow product is determined by the amount of total glutathione.

### Statistical analysis

All data were represented as mean ± SEM. Statistical analysis by two-way ANOVA with Tukey-test post hoc analysis was used in electrophysiology examinations and the training period of the Morris Water Maze. One-way ANOVA with Tukey-test post hoc analysis was for all other studies. The estimation of mixed effect model was used in statistical analysis of the number of spines. The experimental animals were set as the fixed effect, while the different slices in the same animal were set as the random effect. After eliminating the type I error from the random effect, the data had been analyzed by one-way ANOVA. Data were analyzed and figures were generated using the Origin 8.0 software (OriginLab Corporation, USA).

## Results

### Flow chart of experimental procedures and characterization of fullerenol

**Fig. 1:**
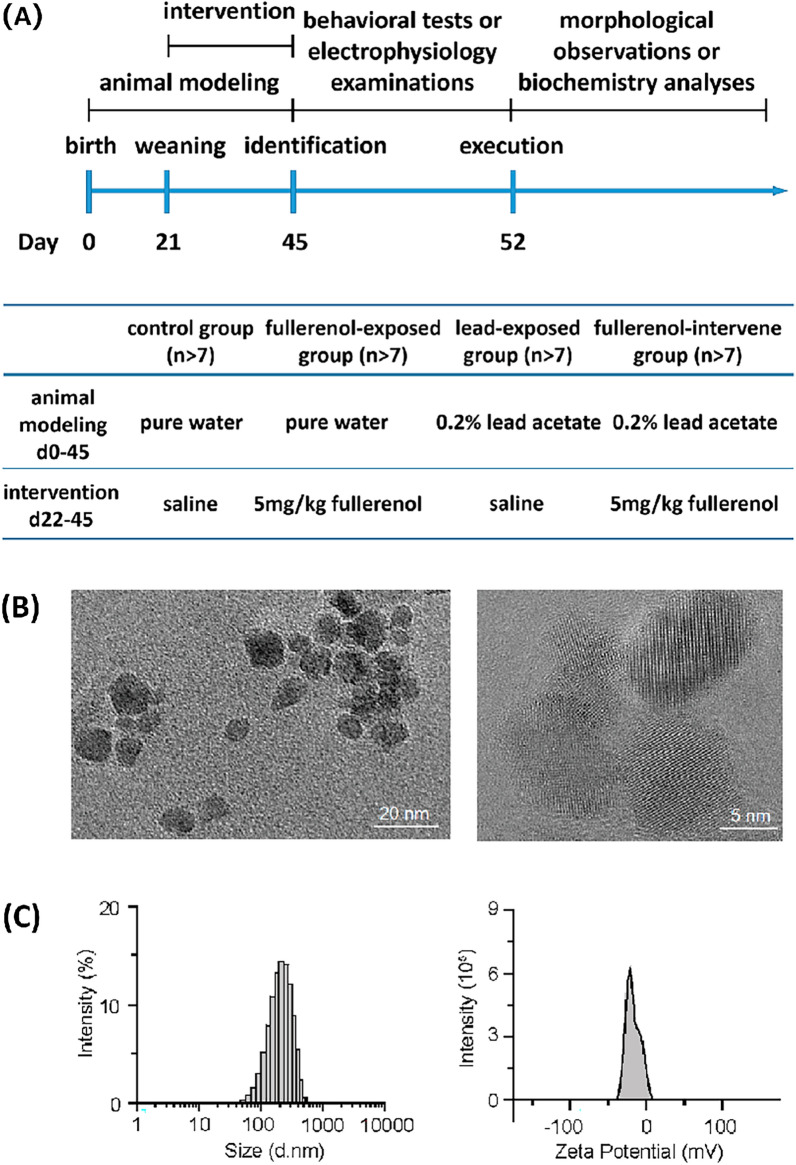
Schematics of the experimental procedures and characterization of fullerenol. **A** Experimental timeline, **B** Transmission electron microscope image, **C** Size distribution and zeta potential of fullerenol (1 μM, dissolved in water)

The overall experimental study design was illustrated in Fig. 1A, including information about the timeline of the procedures. Water-soluble fullerenol was difficult to be characterized because of its amorphous structure. Fullerenol exhibited a circular or rectangular morphology with particle size of about 100 nm as revealed by TEM (Fig. 1B). It was found that the size distribution of single phase fullerenol obeyed normal distribution, and the surface zeta potential was -24.98 ± 3.85 mV (Fig. 1C).

### Identification of fulleronol on animal models

**Fig. 2:**
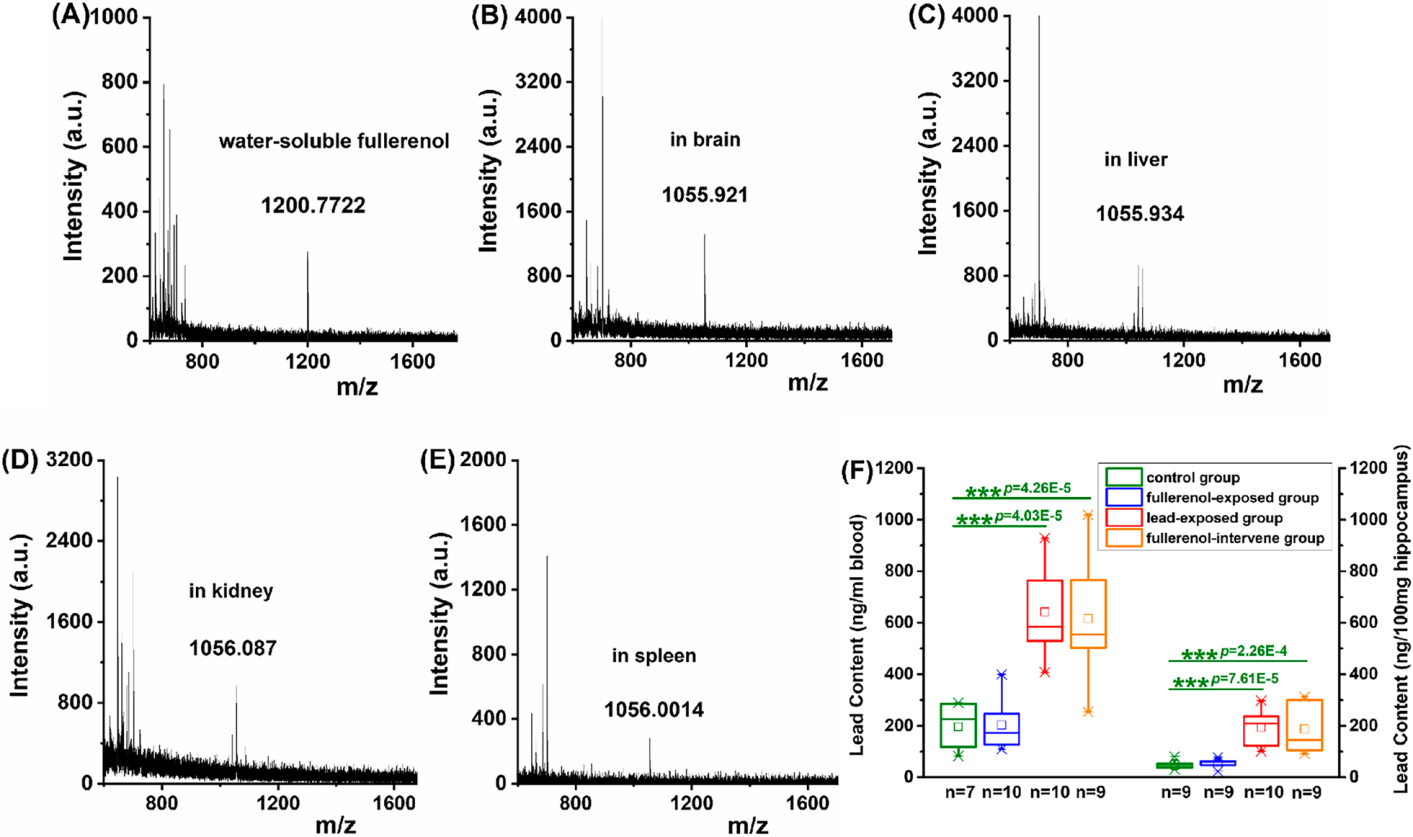
Fullerenol may across the blood–brain barrier and lead can accumulate in the body. **A** Mass spectrometry of water-suspended fullerenol, **B–E** Distribution of exogenous fullerenol in brain, Liver, Kidney and spleen were detected by MALDI-TOF–MS. **F** Lead contents in blood and hippocampus of experiment rats were analyzed by ICP-MS. MALDI-TOF–MS: Matrix-Assisted Laser Desorption/Ionization Time-Of-Flight Mass Spectrometry; ICP-MS: Inductively Coupled Plasma Mass Spectrometry. One-way ANOVA analysis with Tukey-test post hoc analysis. *** *p* < 0.001 compared with the control group

The biological distribution of fullerenol in a rat (i.p., 5 mg/kg) was detected by MALDI-TOF–MS. The mass peak of water-soluble fullerenol [C_60_(OH)_24_] after acidification and purification was at m/z 1200 [M + 4H_2_O] (Fig. 2A), while in the main tissues (brain, liver, kidney and spleen) the most abundant ion might be centered at m/z 1055 [M-4H_2_O-H]^−^ and corresponded to a singly charged ion generated through fullerenol (Fig. 2B-E), indicating that fullerenol might widespread distribute and accumulate in important organs within the body by passing the blood–brain barrier[21]. Compared with rats without lead treatment, the lead content in blood (Total df = 35, F = 19.43, *p* < 0.001) and hippocampus (Total df = 36, F = 15.72, *p* < 0.001) of lead-exposed rats had increased significantly, proving that an animal model of chronic lead poisoning had been successfully made (Fig. 2F).

### Fullerenol improved hippocampus-dependent spatial learning and memory

**Fig. 3:**
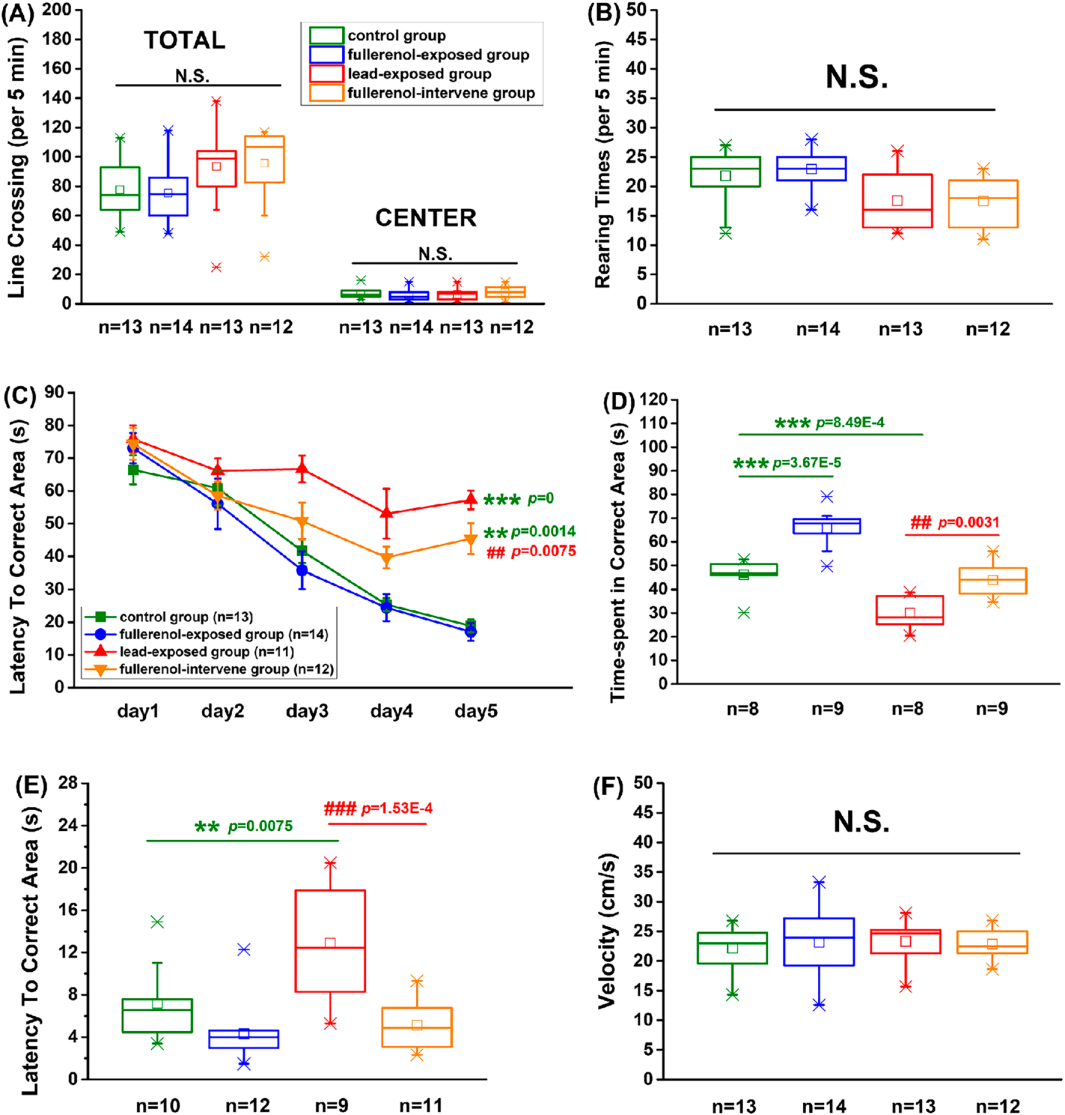
Fullerenol improved hippocampus-dependent spatial learning and memory. **A**, **B** The number of crossing squares and rearing in open field test indicated that exploratory activity of rats were normal. **C–F **The performances of rats in Morris water maze, including latency to the correct platform during training period, time-spent in the correct area, latency to the correct area and velocity, indicated that fullerenol improved the learning and memory. Two-way ANOVA with Tukey-test post hoc analysis was used in the training period of the Morris Water Maze. One-way ANOVA with Tukey-test post hoc analysis was for all other studies. ** *p* < 0.01, *** *p* < 0.001 compared with the control group. ^##^ *p* < 0.01, ^###^ *p* < 0.001 compared with the lead-exposed group. N.S. not significant

In open field test, the number of crossing squares (Total df = 51, F = 2.32, *p* > 0.05; Total df = 51, F = 0.54, *p* > 0.05) and rearing (Total df = 42, F = 3.92, *p* > 0.05) were not significantly changed by fullerenol and lead (Fig. 3A-B), which strongly indicating that the exercise capacity and exploratory activity of rats after various treatments were normal.

In Morris water maze test, lead-exposed animals presented a longer latency to find the platform than non-lead-exposed rats during the training period (Corrected Total df = 222, Model F = 15.11, *p* < 0.001, Fig. 3C). During the testing period, compared with control rats, lead-exposed rats spent less time in the correct quadrant (Total df = 33, F = 33.67, *p* < 0.001, Fig. 3D) and took more time to find the correct area (Total df = 41, F = 11.14, *p* < 0.01, Fig. 3E). However, it was improved in the fullerenol-intervene group (*p* < 0.01 and *p* < 0.001 compared with the lead-exposed group, Fig. 3D, E). Meanwhile, there was no significant difference in the average swimming speed among each group (Total df = 51, F = 0.22, *p* > 0.05, Fig. 3F). These results showed that fullerenol could improve hippocampus-dependent cognition and protect rats against lead-exposure induced impairment in spatial learning and memory.

### Fullerenol enhanced long-term synaptic plasticity in the hippocampus

**Fig. 4:**
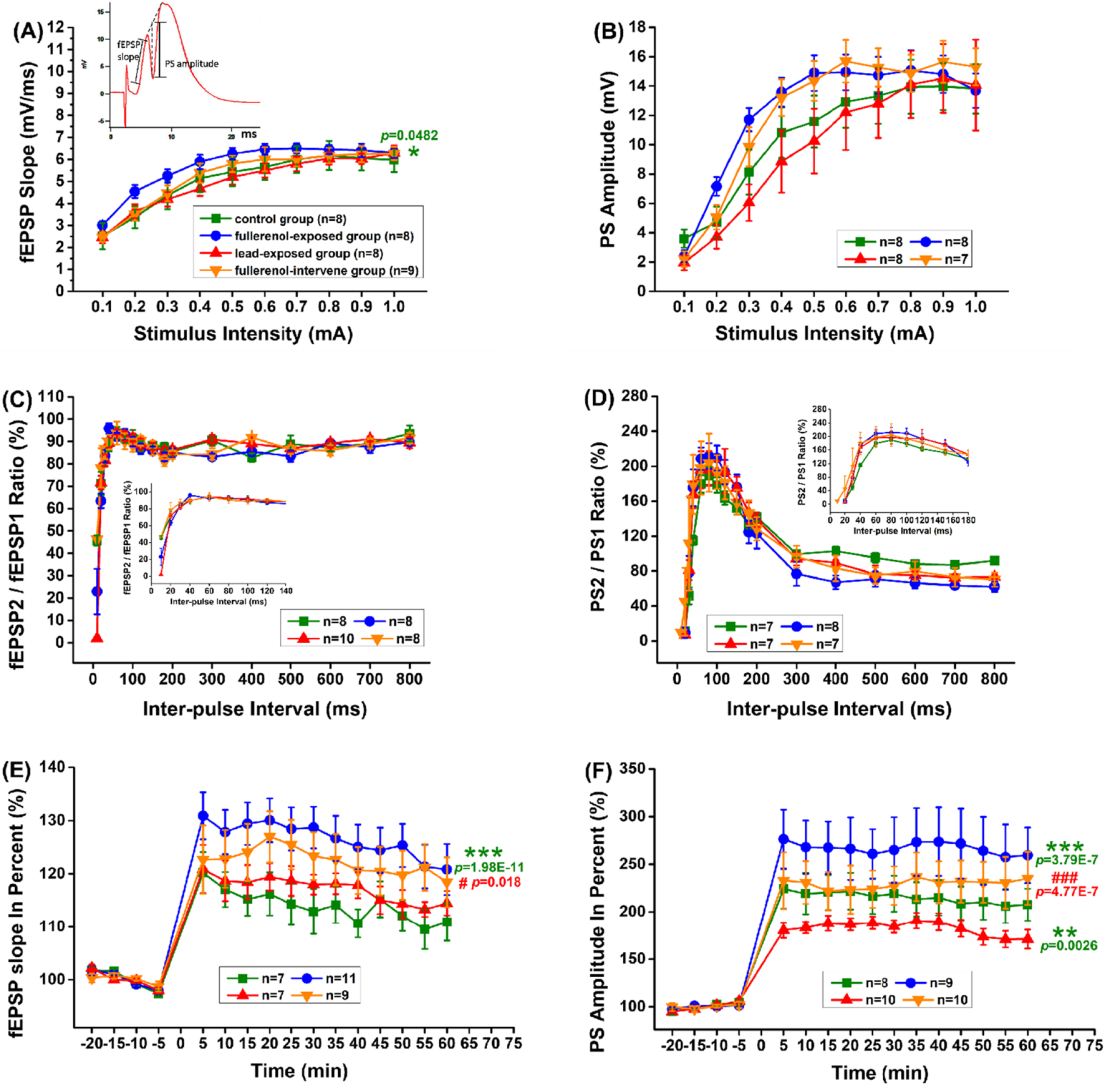
Fullerenol enhanced long-term synaptic plasticity in hippocampal PP-DG pathway. **A, B **The I/O curve shown as fEPSP slope and PS amplitude, **C**, **D** The PPF ratio shown as fEPSP2/fEPSP1 and PS2/PS1, **E**, **F** The LTP induction shown as fEPSP slope in percent and PS amplitude in percent indicated that long-term maintenance of synaptic plasticity by fullerenol was found in hippocampal DG area. One-way ANOVA with Tukey-test post hoc analysis was used in the peak analysis of the PPF ratio curve. Two-way ANOVA with Tukey-test post hoc analysis was for all other studies. * *p* < 0.05, ** *p* < 0.01, *** *p* < 0.001 compared with the control group. ^#^ *p* < 0.05, ^###^ *p* < 0.001 compared with the lead-exposed group

The schematic diagram is in the upper left corner of Fig. 4A. fEPSP slope is calculated as the slope of the first positive wave peak. And PS amplitude is calculated as the distance between the wave bottom and the midpoint of the crest line of the two positive peaks. As shown in Fig. 4A-B, the I/O curve corresponding to fEPSP slope was enhanced in the fullerenol-exposed group (Corrected Total df = 314, Model F = 26.61, *p* < 0.05), while PS amplitude had a slightly increasing trend in both of the two fullerenol treatment group (Corrected Total df = 294, Model F = 21.89, *p* > 0.05). In follow-up experiments, the stimulation intensity was adjusted to evoke potentials that 50% of the maximal PS amplitude, expressed as 0.4 mA.

As shown in Fig. 4C, D, the PPF ratio determined using the measurement of fEPSP2/fEPSP1 and PS2/PS1 had no obvious changes among groups. In addition, as shown in Fig. 4E, fEPSP slope after HFS in the fullerenol-exposed group (126.59 ± 0.94%) was higher than that in the control group (114.03 ± 0.88%, Corrected Total df = 511, Model F = 27.31, *p* < 0.001). Meanwhile, in Fig. 4F, PS amplitude was increased in the fullerenol-exposed group (266.95 ± 1.73%, Corrected Total df = 591, Model F = 32.49, *p* < 0.001) and decreased in the lead-exposed group (182.4 ± 2.05%, *p* < 0.01) by comparing with the control group (215.1 ± 1.73%). Importantly, the suppression in the lead-exposed group vanished in the fullerenol-intervene group (fEPSP slope: 122.38 ± 0.7%, *p* < 0.05; PS amplitude: 229.75 ± 1.36%, *p* < 0.001; compared with the lead-exposed group). These results indicated that fullerenol could enhance synaptic plasticity in the DG area and alleviate the damage caused by lead toxicity to some degree.

**Fig. 5:**
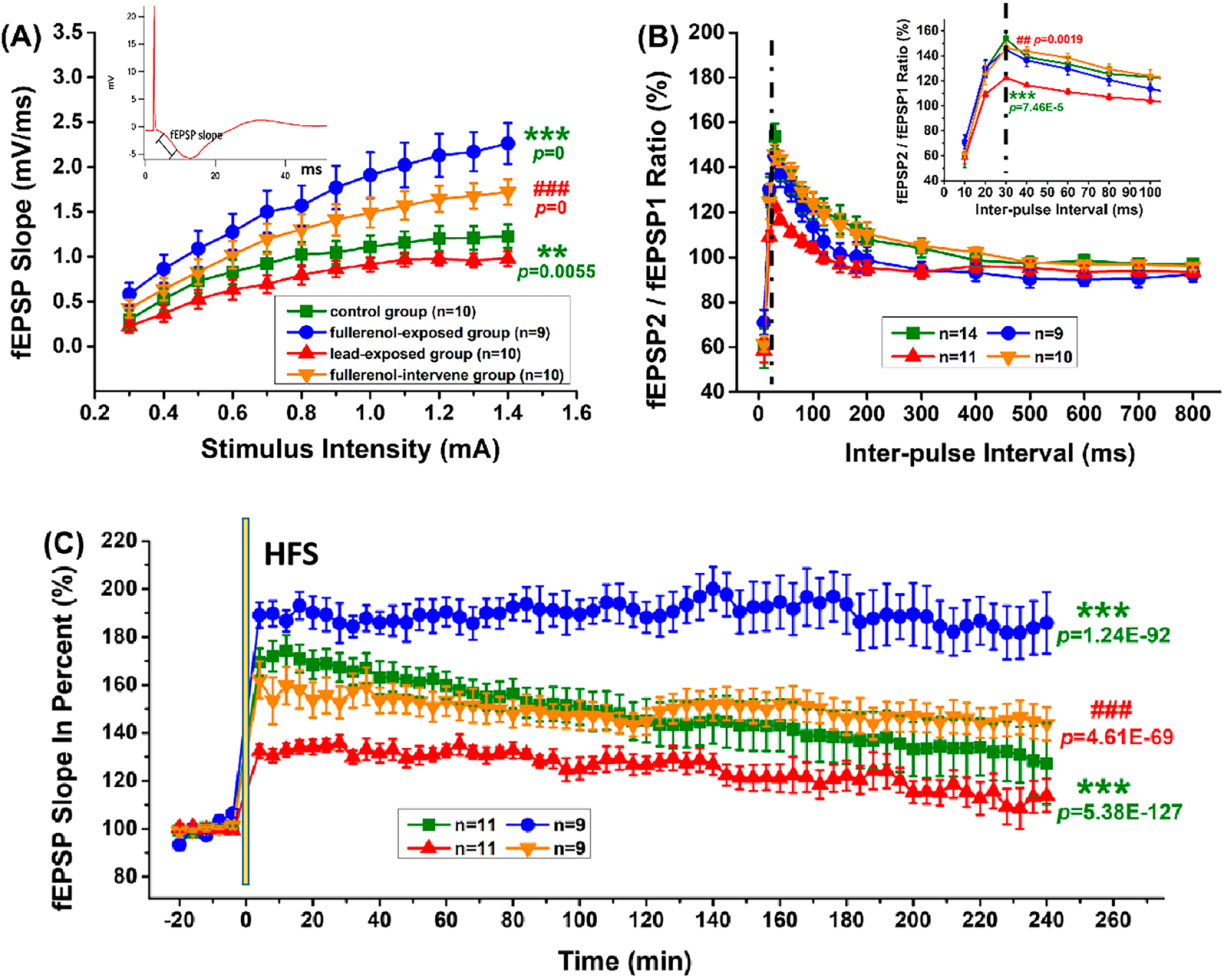
Fullerenol enhanced long-term synaptic plasticity in hippocampal Sch-CA1 pathway. **A **The I/O curve shown as fEPSP slope, **B** The PPF ratio shown as fEPSP2/fEPSP1, **C **The LTP induction shown as fEPSP slope in percent indicated that long-term maintenance of synaptic plasticity by fullerenol was found in hippocampal CA1 area. One-way ANOVA with Tukey-test post hoc analysis was used in the peak analysis of the PPF ratio curve. Two-way ANOVA with Tukey-test post hoc analysis was for all other studies. ** *P* < 0.01, *** *P* < 0.001 compared with the control group. ^##^ *P* < 0.01, ^###^ *P* < 0.001 compared with the lead-exposed group

EPSP slope is calculated as the slope of the positive wave peak, the schematic diagram is in the upper left corner of Fig. 5A. Additionally, as shown in Fig. 5A, the basal synaptic transmission in the Sch-CA1 pathway was decreased by lead (Corrected Total df = 467, Model F = 35.65, *p* < 0.01) and increased by fullerenol (*p* < 0.001), as manifested by in the shift of the I/O function curve. In PPF and LTP experiments, the intensity of single pulses evoking 50% of the maximal PS amplitude was expressed as 0.6 mA.

As shown in Fig. 5B, compared with the control group (150.45 ± 4.88%), the peak of the PPF ratio curve was significantly reduced in the lead-exposed group (122.48 ± 1.13%, Total df = 42, F = 9.43, *p* < 0.001) and rescued in the fullerenol-intervene group (145.83 ± 4.25%, *p* < 0.01, compared with the lead-exposed group), indicating that the lead-induced short-term depression could be altered by fullerenol. Furthermore, as shown in Fig. 5C, the fEPSP slope after HFS was abated by lead (125.32 ± 0.91%, Corrected Total df = 2271, Model F = 41.35, *p* < 0.001, compared with the control group 148.4 ± 1.65%) and raised by fullerenol (189.63 ± 0.52%, *p* < 0.001), while the reduction due to lead disappeared in the fullerenol-intervene group (149.83 ± 0.56%, *p* < 0.001). Notably, this maintenance of synaptic plasticity was a long-lasting strengthening, and lasted at least for 4 h. These results indicate that long-term enhancement of synaptic efficacy occurred in the CA1 area, and fullerenol could change lead-impaired synaptic transmission in the hippocampus.

### Fullerenol altered the PSD-dependent structure in hippocampal CA1 primary neurons

**Fig. 6:**
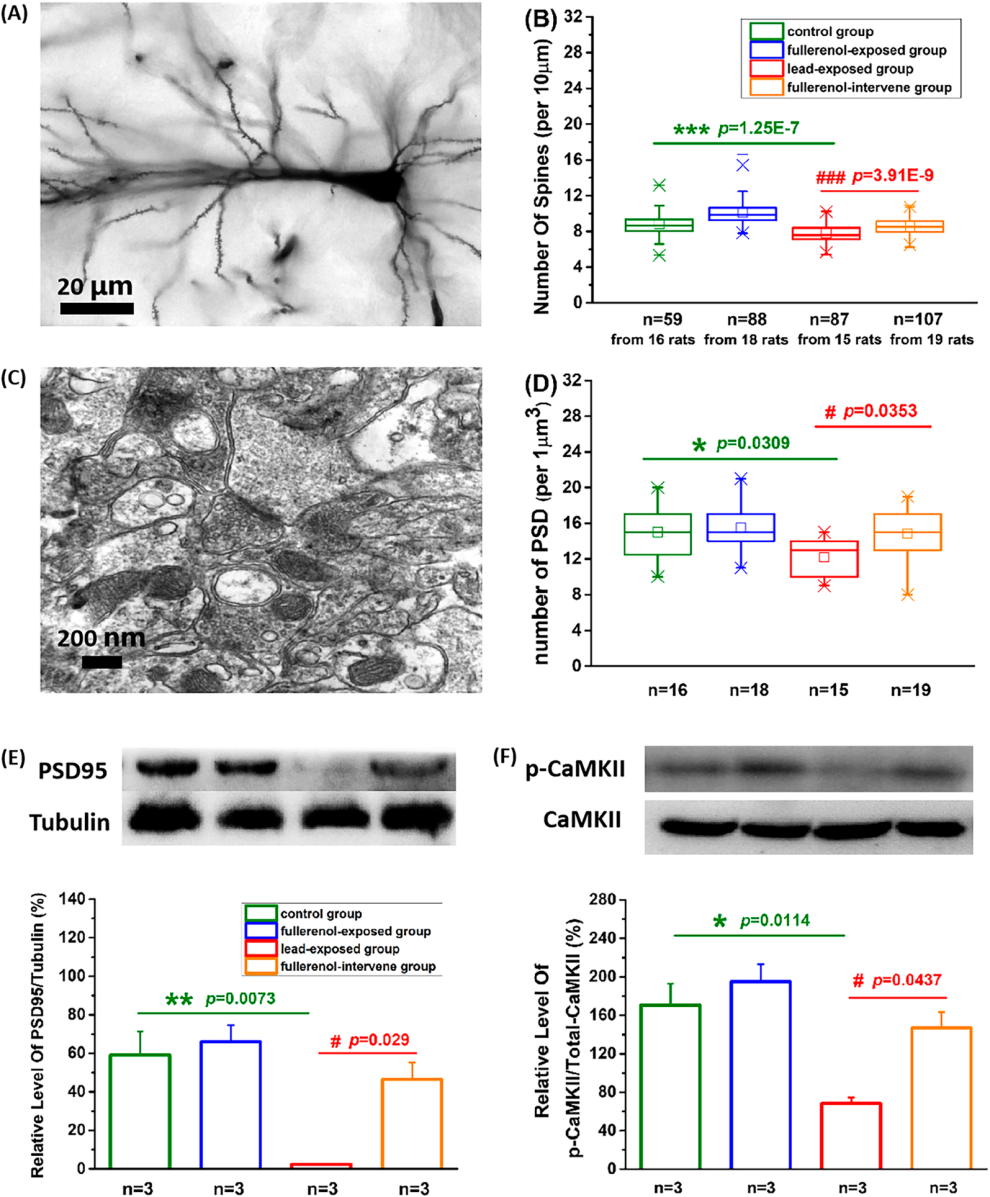
The PSD-dependent structural alteration by fullerenol at the synapse in hippocampus. **A, B** Representative image and statistical analysis of the number of spines, **C, D** The number of PSD, **E, F** Western blot and statistical analysis of PSD95 protein, p-CaMKIIα/total CaMKII showed that fullerenol up-regulated the PSD-dependent structures. One-way ANOVA with Tukey-test post hoc analysis. The estimation of mixed effect model used in statistical analysis of the number of spines eliminated the type I error from the random effect. Date were represented as mean ± SEM. * *p* < 0.05, ** *p* < 0.01, *** *p* < 0.001 compared with the control group. ^#^ *p* < 0.05, ^###^ *p* < 0.001 compared with the lead-exposed group

In Fig. 6A, using Golgi staining, dendritic spines were clearly marked and the number of the second branches of dendrites was accurately counted. The mixed effect model was used, while the different slices in the same animal were set as the random effect. The significant value of the random effect was 0.945, and it eliminated the type I error. In Fig. 6B, compared with the control group (8.64 ± 0.11/10 µm), the number was significantly decreased in lead-exposed group (7.81 ± 0.08/10 µm, Total df = 626, Model F = 147.02, *p* < 0.001), and this trend had been different in the fullerenol-intervene group (8.52 ± 0.06/10 µm, *p* < 0.001, compared with the lead-exposed group). The dendritic spines of neurons are closely related to the formation of PSD plaques. In the center of Fig. 6C, PSD were clearly visible under the electron microscope, and the number of PSD in the lead-exposed group (12.2 ± 0.49/1µm^3^) was significantly less than that in the control group (15 ± 0.77/1µm^3^, Total df = 67, Model F = 4.55, *p* < 0.05), but this decrease was significantly attenuated in the fullerenol-intervene group (14.84 ± 0.73, *p* < 0.01, compared with the lead-exposed group, Fig. 6D). Meanwhile, in Fig. 6E, the level of PSD95 protein was similar to the above quantitative change (Total df = 11, Model F = 10.88). Calcium/calmodulin-dependent protein kinase II (CaMKII) is implicated in LTP, and some studies have shown that there is an increase in CaMKII activity directly in the PSD of dendrites after LTP induction. In Fig. 6F, exposure to lead significantly reduced the pCaMKIIα/CaMKIIα ratio (Total df = 11, Model F = 10.59, *p* < 0.05), but the reduction induced by lead disappeared in the fullerenol-intervene group (*p* < 0.05, compared with the lead-exposed group). These findings indicated that the treatment with fullerenol in our study did up-regulate the number of dendritic spines, the level of PSD95 and the activity of CaMKIIα, which might enhance synaptic efficacy.

### This protective effect of fullerenol was independent on the reduction–oxidation pathway

**Fig. 7:**
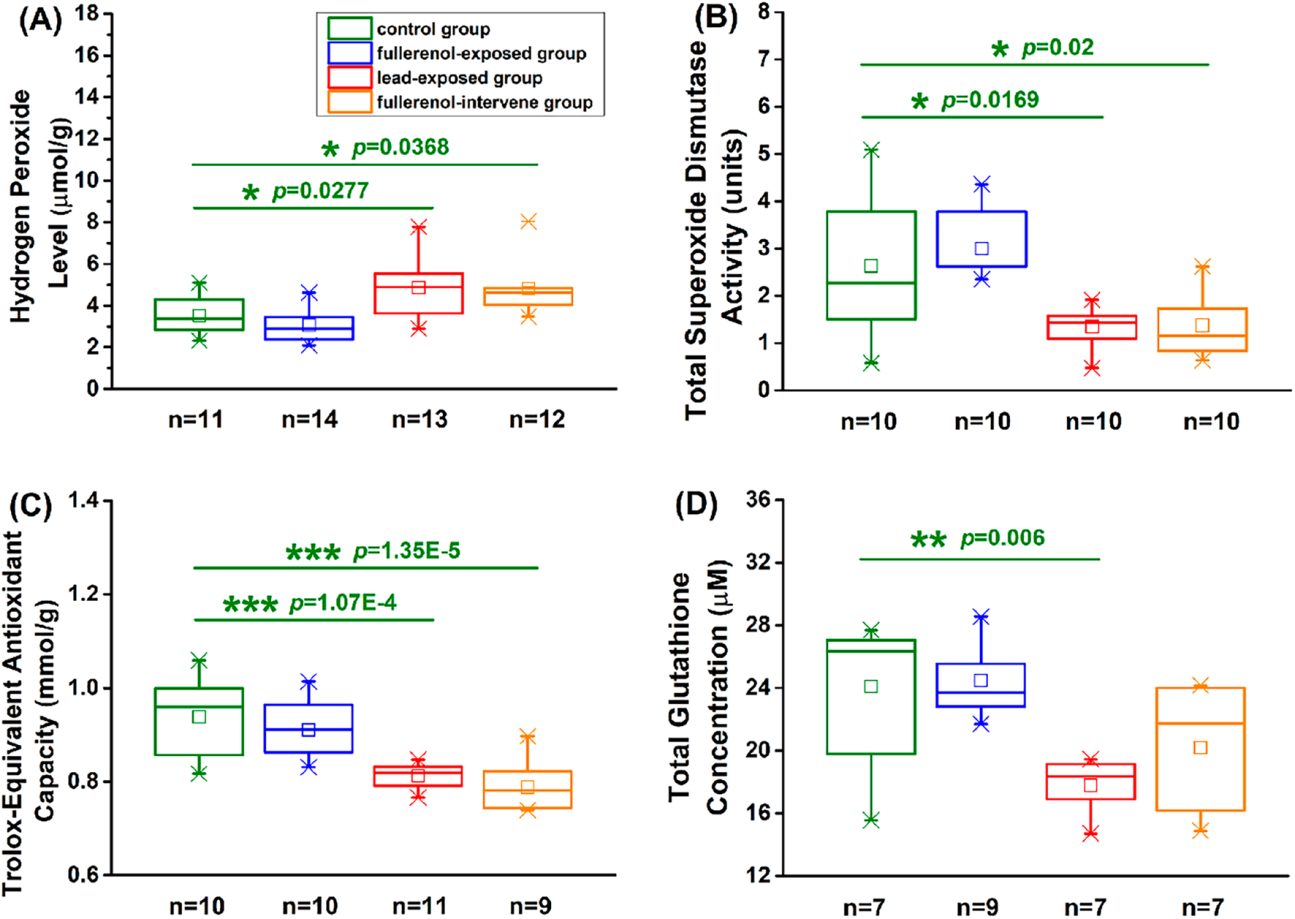
The protective effect of fullerenol was not dependent on the reduction–oxidation pathway. **A** The H_2_O_2_ level. **B **The total SOD activity. **C** The total antioxidant capacity. **D** The total GSH concentration. One-way ANOVA with Tukey-test post hoc analysis. * *p* < 0.05, ** *p* < 0.01, *** *p* < 0.001 compared with the control group

According to the previous study using cultured cells, the protective effect of fullerenol was associated with the redox level [20]. In order to assess the in vivo protective effect of fullerenol, we determined the redox state in hippocampal tissues. As shown in Fig. 7, we found that the level of H_2_O_2_ increased from 3.529 ± 0.257 μmol/g in the control group to 4.86 ± 0.387 μmol/g in the lead-exposed group (Total df = 49, F = 8.59, *p* < 0.05), while the total SOD activity, the total antioxidant capacity and the total GSH concentration decreased from 2.63 ± 0.47 units, 0.94 ± 0.03 mmol/g and 24.09 ± 1.74 μM in the control group to 1.37 ± 0.22 units, 0.81 ± 0.01 mmol/g and 17.79 ± 0.65 μM in the lead-exposed group (Total df = 39, F = 8.76, *p* < 0.05, Total df = 39, F = 15.47, *p* < 0.001; Total df = 29, F = 7.41, *p* < 0.01). However, there were no significant changes between the lead-exposed group and the fullerenol-intervene group (*p* > 0.05). These results indicated that potentiation of spatial learning and memory by fullerenol might be not dependent on the reduction–oxidation pathway.

## Discussion

### An innovative strategy for biological distribution analysis using MALDI-TOF–MS confirms that fullerenol can across the blood–brain barrier.

To study the biological reactions of fullerenol, an in vivo animal model is used in the present study. It is worthy to mention that the concentration-dependent effects of fullerenol have been manifested in our previous research, which is taken form of improving the survival rate of cultured rat hippocampal neurons at lower concentrations of fullerenol [20]. The in vivo performance of a low dose of fullerenol (5 mg/kg, i.p.) in lead-poisoned rats is a sequel study.

The biological distribution of fullerenol detected by MALDI-TOF–MS indicates that fullerenol may across the blood–brain barrier and accumulate in the brain. Fullerenol C60(OH)24, as a large molecular weight and electrically neutral compound, is very suitable for MALDI-TOF–MS [21]. Using this high-resolution technique, some researchers had mapped the distribution of fullerenols in zebrafish tissues [22]. Herein, we are happy to report that we find a deprotonated molecule plus loss of HO` and/or H` radicals [M-HO`-H`-H]− in important organs including brain. This deprotonated molecule observed in MALDI-TOF–MS spectra of fullerenol again demonstrates that the nervous system may be the target organ and fullerenol may be described as a new kind of nanomedicine.

### The in vivo amelioration effect of fullerenol in a rat model of lead exposure exhibits good therapeutic potential.

Using behavioral tests, we find the in vivo performance of fullerenol can affect lead-induced-impaired learning and memory. Lead can interfere with the activities of several protein and gene expressions, so it is identified as a highly poisonous heavy metal affecting several organ systems in the body [23]. Nowadays, lead poisoning and lead pollution are mainly treated by eliminating lead [24, 25]. From the perspective of repairing the functions of nervous system, our paper adds a creative idea of treating lead poisoning.

At present, other recent articles are focusing on the transportation potential of nanomedicines as drug-carrier materials [26]. Our research shows that fullerenol as a drug carrier has an obvious repairing effect on lead-poisoning, which exhibits good therapeutic potential. The non-toxic and therapeutic feature of fullerenol will be beneficial to the discovery and development as a new drug and the biological application as a drug carrier.

### Long-term synaptic plasticity plays a vital role in mitigating the negative effects of lead on nervous system.

In order to explore the possible mechanism of fullerenol in protecting hippocampus from being injured by lead, in vivo extracellular field potential recording has been used in our study. Unlike in vitro intracellular electrophysiology recording, it may be more truly and completely reflect the efficacy of drugs, through some important indicators such as I/O function, PPF ratio and LTP [27, 28]. Two major pathways into and out of the hippocampus (the PP-DG pathway and the SCh-CA1 pathway), coordinately maintain synaptic transmission in hippocampus and are suitable to verify the possible effect of synaptic plasticity in the process of fullerenol against lead-induced injury [29–31]. Our results show that fullerenol has the positive impacts on hippocampal plasticity, and can eliminate the inhibitory effect of lead. Especially in the formation of LTP in the SCh-CA1 pathway, we continue to record for 4 h after HFS. It is widely recognized that enhancement lasting longer than 2–3 h is believed to require synthesis of new proteins [32, 33]. A recent article has shown that fullerenols have neuroprotective activities, including promoting restoration of dopamine levels, reducing oxidative stress, preventing death of neurons and alleviating aggregation of alpha-synuclein [34]. But our previous study of fullerenol on synaptic plasticity in hippocampal brain slices of rats indicates that high-level fullerenols depress the activity and the expression of nitric oxide synthase in hippocampus [35]. The role of fullerenols in the nervous system is controversial. Our studies in this paper imply an inference that stabilizing synaptic plasticity and optimizing synaptic architecture are necessary in the role of fullerenol in mitigating the negative effects of lead on nervous system.

### The PSD-dependent transition of synaptic potentiation to structural alterations in hippocampus may underlie neuroprotective effects of fullerenol.

As we all know, changes in the number and structure of the synapse can cause changes in synaptic plasticity, thereby affect learning and memory [36]. In the present study, structural accommodations and functional adjustments of hippocampal neurons are almost the same, while the morphological results show that fullerenol can increase the number of dendritic spines and PSD In addition, the level of PSD95 protein in hippocampal CA1 areas, as an important component of PSD, has also confirmed this result. A great deal of evidence demonstrates that the activation of CaMKII is essential and sufficient in the process of LTP induction and memory formation [37, 38]. It provides a strong evidence to support our aforesaid morphological results according to our observation that the level of activated CaMKII through auto-phosphorylation at T286 is persistently elevated after fullerenol treatment, while the level of total CaMKII remains relatively unchanged. In general, most studies use electrophysiological techniques to analyze learning and memory, but this research adds morphological and molecular assessments on the basis, and then it is found that enhancing synaptic efficacy accompany the performance of these PSD-dependent structural alterations and new synthesized proteins. However, this repair effect of fullerenol may not be directly related to the redox state at the concentration used in this studyAbundant evidences indicate that oxidative stress plays a critical role in cognitive functions, including hippocampal synaptic plasticity [39–42]. In the present study, rather than repairing lead-injured rats through the redox ability, fullerenol may directly improve the learning and memory in normal or injured rats by generating and maintaining synaptic plasticity. Have to admit that further studies are needed to determine the pathway leading to the in vivo effects of fullerenol on the impaired hippocampus.

## Conclusion

Fullerenol has broad potential applications in biology and medicine because of its highly water soluble, when there are few studies focusing on the in vivo roles of fullerenol in learning and memory. And the deprotonated molecule observed in MALDI-TOF–MS spectra of fullerenol again demonstrates that the nervous system can be the target organ. The study has shown that fullerenol can ameliorate lead-induced-impaired learning and memory in vivo, due to long-term maintenance of synaptic plasticity which is showed as enhanced synaptic efficacy and additional structural alteration. The possible mechanism is shown in additional Fig. S1. It provides a theoretical basis for evaluating the biological effects of fullerenol in the nervous system and lays a solid foundation for further discovery and development as a new nanomedicine.

## Supplementary Information

**Additional file 1. Fig. S1. **A schematic diagram of mechanism of fullerenol ameliorating lead-induced-impaired learning and memory.

## Data Availability

The datasets used and/or analysed during the current study are available from the corresponding author on reasonable request.
