# Supplementary figures and images for: Long-term maintenance of synaptic plasticity by Fullerenol Ameliorates lead-induced-impaired learning and memory in vivo
Source: J Nanobiotechnology. 2022 Aug 1;20:348. doi: 10.1186/s12951-022-01550-2 (PMC9341061; doi:10.1186/s12951-022-01550-2)

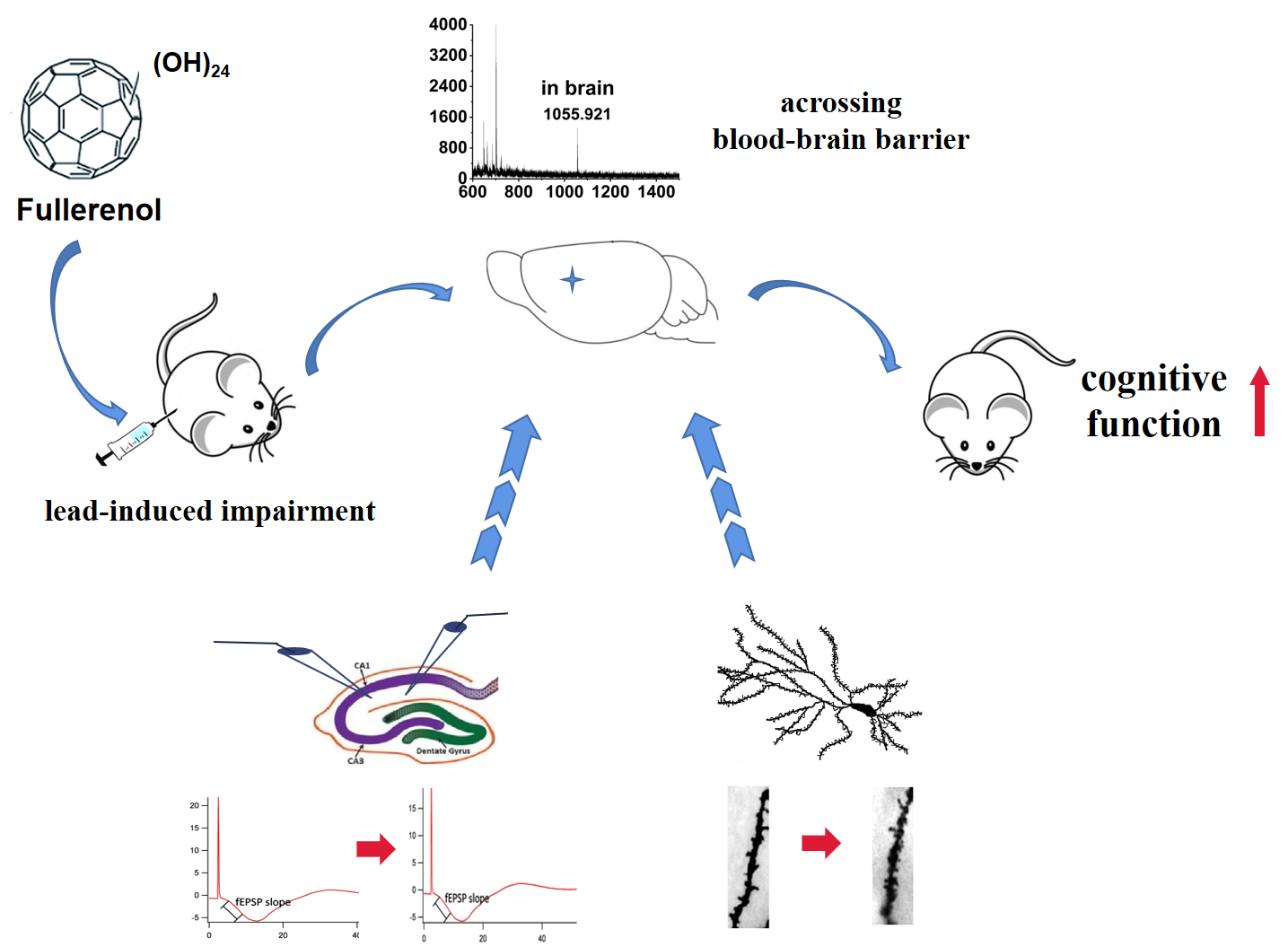

Supplement: Supplementary file 1 — Additional file 1. Fig. S1. A schematic diagram of mechanism of fullerenol ameliorating lead-induced-impaired learning and memory. [file 12951_2022_1550_MOESM1_ESM.tif]
